# Comparing Lenvatinib/Pembrolizumab with Atezolizumab/Bevacizumab in Unresectable Hepatocellular Carcinoma: A Real-World Experience with Propensity Score Matching Analysis

**DOI:** 10.3390/cancers16203458

**Published:** 2024-10-12

**Authors:** Yu-Chun Hsu, Po-Ting Lin, Wei Teng, Yi-Chung Hsieh, Wei-Ting Chen, Chung-Wei Su, Ching-Ting Wang, Pei-Mei Chai, Chen-Chun Lin, Chun-Yen Lin, Shi-Ming Lin

**Affiliations:** 1 Department of Gastroenterology and Hepatology, Chang Gung Memorial Hospital, Linkou Branch, Taoyuan 333, Taiwan; tony2005pc@gmail.com (Y.-C.H.);; 2 College of Medicine, Chang Gung University, Taoyuan 333, Taiwan; 3 Graduate Institute of Clinical Medical Sciences, College of Medicine, Chang Gung University, Taoyuan 333, Taiwan; 4 Department of Nursing, Chang Gung Memorial Hospital, Linkou Branch, Taoyuan 333, Taiwan; 5 Department of Gastroenterology and Hepatology, New Taipei Municipal Tucheng Hospital, New Taipei 236, Taiwan

**Keywords:** hepatocellular carcinoma (HCC), lenvatinib, pembrolizumab, atezolizumab, bevacizumab

## Abstract

**Simple Summary:**

Systemic therapy is the primary treatment option for patients diagnosed with unresectable hepatocellular carcinoma. The aim of our retrospective study is to assess the comparative effectiveness and safety of two treatment regimens in the first-line setting: lenvatinib plus pembrolizumab and atezolizumab plus bevacizumab. Our findings indicate that both regimens show similar overall survival, progression-free survival, and acceptable safety profiles in real-world conditions.

**Abstract:**

Background: The combination of anti-angiogenic therapy and immune checkpoint inhibitors has revolutionized the management of unresectable hepatocellular carcinoma (uHCC). While an early-phase study demonstrated promising outcomes for lenvatinib plus pembrolizumab (L+P) in treating uHCC, the LEAP-002 trial did not meet its primary endpoint. However, the comparative efficacy between L+P and atezolizumab plus bevacizumab (A+B) as first-line treatment remains a topic of uncertainty. This study aimed to assess the effectiveness and safety of L+P in contrast to A+B among patients diagnosed with uHCC. Methods: We conducted a retrospective analysis of enrolled patients with uHCC who received L+P or A+B as initial systemic treatment at Chang Gung Memorial Hospital from June 2019 to December 2022. The overall survival (OS), progression-free survival (PFS), objective response rate (ORR), and disease control rate (DCR) by modified RECIST were compared. Results: 121 patients were recruited, with 37 receiving L+P and 84 receiving A+B. Among them, 95 (78.5%) patients were BCLC stage C, and 99 (81.8%) patients had viral etiology for HCC, predominantly chronic HBV (68.6%). Both the L+P and the A+B groups demonstrated comparable OS (18.2 months versus 14.6 months, *p* = 0.35) and PFS (7.3 months versus 8.9 months, *p* = 0.75). The ORR and DCR were similar. After propensity score matching, the results remained consistent between the matched patients. Treatment-related adverse events of any grade occurred in 30 (81.1%) in the L+P group and 62 (73.8%) in the A+B group. Conclusions: Our findings suggest that L+P and A+B exhibit comparable efficacy and safety profiles in real-world settings.

## 1. Introduction

Primary liver cancer is the third leading cause of global cancer death in 2020, with the majority of cases being hepatocellular carcinoma (HCC) [1]. Systemic therapy is currently the main treatment option for patients with unresectable HCC, including patients with advanced-stage HCC and selected patients with intermediate-stage HCC [2,3]. Since the phase 3 SHARP trial, sorafenib, an oral multi-kinase inhibitor (MKI), became the backbone of the first-line treatment for advanced-stage HCC [4]. The phase 3 REFLECT trial demonstrated that lenvatinib, also an MKI, was non-inferior to sorafenib in overall survival [5]. Early phase studies showed pembrolizumab and nivolumab, both programmed death 1 (PD-1) inhibitors, had promising antitumor activity and manageable safety in patients with advanced HCC previously treated with sorafenib [6,7]. Subsequently, the phase 3 IMbrave150 trial established the role of combination therapy in unresectable HCC. The atezolizumab plus bevacizumab (A+B), a programmed death-ligand 1 (PD-L1) inhibitor plus a monoclonal antibody that blocks vascular endothelial growth factor (VEGF), showed better overall survival and progression-free survival than sorafenib in the primary and the updated analyses [8,9]. Based on these clinical trials, the current guidelines state that atezolizumab plus bevacizumab is one of the preferred first-line treatment options for patients with advanced cancer [2,10,11]. Three recent phase 3 randomized controlled trials (LEAP-002, CARES-310, and COSMIC-312) have examined the efficacy of the antiangiogenic drug plus the immune checkpoint inhibitor (ICI), but they showed mixed results [12,13,14]. Of them, the global, double-blinded LEAP-002 trial compared the lenvatinib plus pembrolizumab (L+P) to lenvatinib monotherapy. L+P did not meet the prespecified dual primary endpoint but achieved a long median overall survival of 21.2 months [13]. 

The real-world experience and outcomes of L+P remain intriguing. In addition, in the era where A+B has become the current standard of care of 1st treatment, it is noteworthy that there are no clinical trials using A+B as the control arm. To investigate the efficacy and safety of L+P in patients with unresectable HCC and to compare outcomes between the L+P and A+B cohorts, we conducted this real-world study.

## 2. Materials and Methods

### 2.1. Study Design and Patients

Patients receiving lenvatinib plus pembrolizumab or atezolizumab plus bevacizumab at our institute, Chang Gung Memorial Hospital, from June 2019 to December 2022 were retrospectively enrolled. These patients were followed up until August 2023. HCC was confirmed by pathological samples or dynamic computed tomography (CT) or magnetic resonance imaging (MRI) according to AASLD guidance [2]. We included patients who met all of the following inclusion criteria: (1) age > 18 years, (2) diagnosis of unresectable HCC in either the intermediate (BCLC stage B) or advanced stage (BCLC stage C), (3) presence of at least one measurable lesion per modified RECIST criteria [15], and (4) classified as Child–Pugh class A or B. Key exclusion criteria included (1) receiving only one cycle of L+P or A+B, (2) being in the early stage (BCLC stage 0/A) or terminal stage (BCLC stage D), (3) having received prior systemic cancer treatment or (4) having missing clinically relevant data. 

### 2.2. Treatments

In the L+P group, the dose of lenvatinib was determined based on body weight, with patients weighing less than 60 kg receiving 8 mg/day and those weighing 60 kg or more receiving 12 mg/day. Pembrolizumab was planned to be administered at 2 mg/kg intravenously every three weeks. The dose and frequency of lenvatinib could be adjusted based on drug intolerance. In the A+B group, atezolizumab 1200 mg plus bevacizumab 15 mg/kg was administered intravenously every three weeks. The dose and frequency of regimens could be adjusted based on drug intolerance or economic considerations. Patients continued to receive the combination therapy until radiological or clinical progression, severe adverse events, or based on the patient’s decision.

### 2.3. Assessments

We retrospectively collected clinical variables before systemic treatment, including age, sex, etiology of viral hepatitis, Child–Pugh score, albumin–bilirubin (ALBI) score, alpha-fetoprotein (AFP) level, BCLC stage, portal vein thrombosis stage (Vp1-Vp4), and presence of extrahepatic spread. We adopted the concept of up-to-seven criteria and up-to-eleven criteria for assessing intrahepatic tumor burden [16,17]. Beyond up-to-seven criteria meant the sum of the number of tumors and the size of the largest tumor was greater than seven. Up-to-eleven criteria were utilized with similar concept as up-to-seven criteria with a sum greater than eleven. 

We evaluated tumor response by CT or MRI every 2–3 months. The treatment response was classified into complete response (CR), partial response (PR), stable disease (SD), and progressive disease (PD) according to modified RECIST criteria (mRECIST). The primary endpoint of the present study was overall survival (OS); the secondary endpoints were progression-free survival (PFS), objective response rate (ORR), and disease control rate (DCR). ORR was defined as the percentage of patients achieving CR and PR; DCR was defined as the percentage of patients achieving CR, PR, and SD. Overall survival (OS) was defined as the period from initiation of combination treatment to death due to any cause. Progression-free survival (PFS) was defined as the period from initiation of the combination treatment to disease progression or death. Early AFP response was defined as baseline AFP level ≥ 10 ng/mL and > 10% reduction within 4 weeks of treatment [18]. The combination of systemic therapy and locoregional therapy was allowed. The locoregional therapies include transarterial chemoembolization, radiofrequency ablation, or radiotherapy targeting liver tumors during systemic therapy. Adverse events and their severity were recorded according to the Common Terminology Criteria for Adverse Events (CTCAE version 5.0).

### 2.4. Propensity Score Matching and Statistical Analyses

In this study, we used propensity score matching (PSM) to reduce the bias and estimate the effect of the treatment. We estimated the propensity score using a logistic regression model and put the following covariates at baseline into the model: age, sex, ALBI grade, presence of viral etiology, BCLC stage, presence of Vp4 portal vein thrombosis, beyond up-to-seven criteria, AFP greater than 400 ng/mL. We paired the L+P and A+B cohorts using one-to-one propensity score matching with the nearest neighbor matching without caliper. Distribution of propensity score and balance assessment are reported in Supplement.

Before and after PSM, we reported the comparison of baseline data, radiologic response, and survival curves between the two groups. ORR and DCR were compared by chi-square test. The Kaplan–Meier method with the log-rank test was applied for the comparison of OS and PFS. Univariate and multivariate Cox proportional hazard models were used to estimate the potential risk factors influencing OS and PFS. The choice of L+P or A+B and clinical variables with univariate *p*-value < 0.05 would be considered in multivariate analyses. A two-tailed *p*-value < 0.05 was considered a statistically significant difference.

All the above statistical analyses were performed using R software (version 4.3.2; R Foundation for Statistical Computing, Vienna, Austria).

## 3. Results

### 3.1. Baseline Characteristics of HCC Patients Receiving Either A+B or L+P Treatment

**Figure 1:**
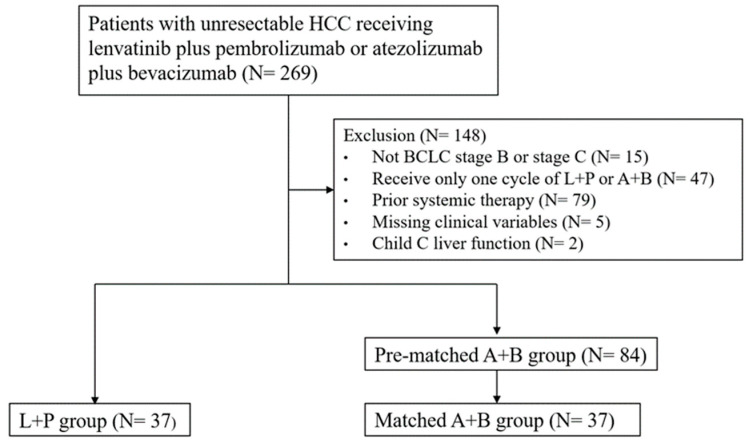
Flowchart of the study design.

In total, there were 269 patients with unresectable HCC who either received L+P (*n* = 80) or A+B (*n* = 189). After excluding the patients who did not fit the inclusion criteria, there were 121 unresectable HCC patients treated with either L+P (*n* = 37) or A+B (*n* = 84) (shown in Figure 1). Baseline demographics and clinical features are presented in Table 1. The mean age was 61.5 years, and the majority (78.5%) were male. A total of 99 (81.8%) patients had viral etiology for HCC. Of them, 83 patients (68.6%) were HBV-related and 20 (16.7%) were HCV-related. A total of 107 (88.4%) patients had Child–Pugh class A liver function, and 48 (39.7%) had ALBI grade I at baseline. A total of 95 (78.5%) patients were classified as BCLC stage C, while others were classified as BCLC stage B. As for the tumor burden, 103 (85.1%) patients had intrahepatic tumors beyond up-to-seven criteria, 30 (24.8%) had Vp4 stage portal vein thrombosis, and 52 (43.0%) had extrahepatic spread (EHS). The median AFP level was 555.4 ng/mL, with 65 (53.7%) patients having AFP levels greater than 400 ng/mL before treatment. There was no statistical difference in the aforementioned parameters between the L+P group and the A+B group.

The dose of L+P or A+B per cycle could be modified by clinicians. The majority (31/37, 83.8%) of patients received pembrolizumab 100 mg per cycle. Additionally, two patients received 50 mg per cycle, two patients received 150 mg per cycle, and two patients received 200 mg per cycle. All patients using A+B received atezolizumab 1200 mg plus bevacizumab 500 mg per cycle. 

### 3.2. Comparing Treatment Efficacy of A+B and L+P before and after Propensity Score Matching

**Figure 2:**
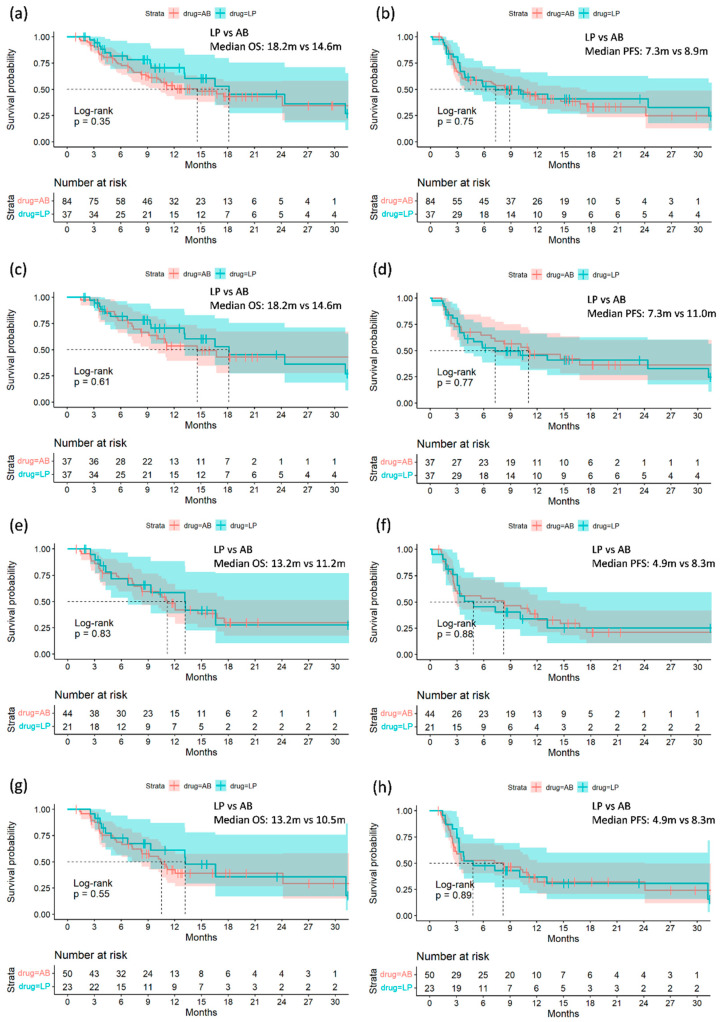
Kaplan–Meier curves of (**a**) overall survival (OS) of the entire cohort; (**b**) progression-free survival (PFS) of the entire cohort; (**c**) OS of the matched cohort; (**d**) PFS of the matched cohort; (**e**) OS of the patients having AFP level > 400 ng/mL at baseline; (**f**) PFS of the patients having AFP level > 400 ng/mL at baseline; (**g**) OS of the patients having intrahepatic tumor burden beyond the eleven-criteria; (**h**) PFS of patients having intrahepatic tumor burden beyond the eleven-criteria.

Both the L+P group and the pre-matched A+B group had similar treatment duration and clinical outcomes (Table 2). The median treatment duration was 2.8 months in the L+P group and 3.2 months in the A+B group. The median duration of follow-up was 9.4 months and 10.1 months, respectively. The ORR in the first evaluation was 30% and 33% (*p* = 0.70), and the DCR was 65% and 63%, respectively (*p* = 0.85). The median PFS was comparable between the L+P group and the A+B group (7.3 months vs. 8.9 months, log-rank *p* = 0.75). Similarly, the median OS was similar between the two groups (18.2 months vs. 14.6 months, log-rank *p* = 0.35) (shown in Figure 2a,b). After PSM, 37 patients received L+P, and 37 patients received A+B. This matched cohort with balanced clinical variables (Table S1). The tumor response was similar between the two subgroups (ORR: A+B vs. L+P: 38% vs. 30%, *p* = 0.47; DCR A+B vs L+P: 73% vs. 65%, *p* = 0.46). The survival outcomes of the L+P patients were comparable to those of the matched A+B patients. The median PFS in the matched A+B group was 11.0 months, compared to 7.3 months in the L+P group (log-rank *p* = 0.77). The median OS in the matched A+B patients was 14.6 months, compared to 18.2 months in the L+P group (log-rank *p* = 0.61) (shown in Figure 2c,d).

### 3.3. Clinical Outcome Predictors in HCC Patients Undergoing A+B and L+P Treatment

The univariate Cox regression analyses of OS revealed several poor prognostic factors in the entire cohort, including Child–Pugh class B (HR 2.21, 95% CI 1.11–4.39), ALBI grade II or III (HR 1.90, 95% CI 1.09–3.31), BCLC stage C (HR 2.33, 95% CI 1.09–5.00), Vp4 portal vein thrombosis (HR 2.32, 95% CI 1.31–4.09), and AFP level > 400 ng/mL (HR 1.77, 95% CI 1.03–3.04). The choice between L+P or A+B did not significantly affect OS (HR 0.75, 95% CI 0.41–1.37). In the multivariate Cox analysis of OS, only Vp4 portal vein thrombosis met statistical significance (HR 2.08, 95% CI 1.17–3.72, *p* = 0.01, Table 3a). In the univariate analyses of PFS, Child–Pugh class B (HR 2.10, 95% CI 1.13–3.93), BCLC stage C (HR 2.02, 95% CI 1.05–3.86), and AFP > 400 ng/mL (HR 1.64, 95% CI 1.02–2.64) predicted poor PFS, while early AFP response (HR 0.40, 95% CI 0.24–0.68) and combining locoregional therapy (HR 0.57, 95% CI 0.35–0.92) predicted better outcome. The choice of treatment regimens did not influence PFS. Child–Pugh class B (HR 2.77, 95% CI 1.45–5.29, *p* = 0.002), early AFP response (HR 0.40, 95%CI 0.24–0.67, *p* < 0.001), and combining locoregional therapy (HR 0.56, 95%CI 0.34–0.92, *p* = 0.022) were the three variables that met statistical significance in the multivariate analysis of PFS (Table 3b). 

### 3.4. Efficacy across the Subgroups

When analyzing the subgroup patients, no survival difference was noted between L+P and A+B. Patients with AFP levels exceeding 400 ng/mL at baseline, L+P, and A+B demonstrated equivalent median PFS (4.9 months vs. 8.3 months, *p* = 0.88) and median OS (13.2 months vs. 11.2 months, log-rank *p* = 0.83). Patients with tumors beyond the up-to-eleven criteria also exhibited comparable median PFS (4.9 months vs. 8.3 months, *p* = 0.89) and median OS (13.2 months vs. 10.5 months, *p* = 0.55) (shown in Figure 2e–h). In patients with Child–Pugh A liver function, L+P, and A+B displayed comparable median PFS (13.1 months vs. 8.9 months, *p* = 0.39) and median OS (24.4 months vs. 16.7 months, *p* = 0.22) (shown in Figure S2).

### 3.5. Comparison of Treatment-Related Adverse Events (TRAE) between Two Treatment Groups

There was no difference in the occurrence of TRAE of any grade between L+P and A+B groups (81.1% vs. 73.8%, *p* = 0.53) (Table 4). Patients receiving L+P experienced a numerically higher percentage of grade 3 TRAEs compared to those receiving A+B (27.0% vs. 19.0%, *p* = 0.46). The common adverse events in the L+P group were proteinuria (35.1%), elevated AST (27.0%), elevated ALT (32.4%), diarrhea (24.3%), palmar-plantar erythrodysesthesia (PPE) (21.6%), anorexia (16.2%), hypertension (13.5%), and skin rash (13.5%). On the contrary, the common adverse events in the A+B patients were elevated AST (38.1%), elevated ALT (27.4%), anorexia (11.9%), proteinuria (11.9%), esophageal variceal bleeding (EVB) (10.7%). The most common severe TRAEs were PPE in the L+P group and EVB in the A+B group. Experiencing TRAEs of grade 3 or higher showed a trend towards worsening OS (HR 1.79, 95% CI 1.00–3.21, *p* = 0.05) and PFS (HR 1.53, 95% CI 0.90–2.59, *p* = 0.12). A higher percentage of patients with Vp4 portal vein thrombosis suffered from high-grade TRAE (VP4 vs without VP4: 36.7 vs. 16.5%, *p* = 0.04). Three patients received L+P with Vp4 thrombosis suffering from high-grade TRAEs (one with EVB requiring urgent intervention, one with PPE and hypertension, and one with immune-related skin adverse events and sepsis). On the other hand, eight patients who received A+B with Vp4 experienced high-grade TRAEs (four with EVB requiring urgent intervention, two with sepsis, one with immune-related skin adverse events, and one with severe encephalopathy). 

## 4. Discussion

Our real-world study demonstrated that both L+P and A+B yielded similar tumor response and survival outcomes for patients with unresectable HCC regardless of tumor burden. Similarly, the adverse effects were also comparable between these two regimens.

In our study, the A+B groups demonstrated numerically higher PFS (8.9 months) to IMbrave150 study (6.9 months) but numerically lower OS (14.6 months) to IMbrave150 study (19.2 months) [8]. As for the L+P groups, our study demonstrated numerically lower PFS (7.3 months) to LEAP-002 trial (8.2 months) and lower OS (18.2 months) to LEAP-002 trial (21.2 months) [13]. The possible reasons for the numerically lower OS and/or PFS in our study might be the disease severity of the patients enrolled. As for tumor burden, the IMBrave150 study enrolled 38% of patients with macrovascular invasion (MVI). The LEAP-002 study enrolled 18% of patients with MVI. In contrast, our study enrolled a higher percentage (55.4%) of patients with MVI. In addition, both clinical trials excluded patients with Child–Pugh B liver function. Our study included 11.6% of patients with Child–Pugh class B. These differences in the disease severity might explain the slightly lower in OS and/or PFS in our study cohort.

Despite the fact that A+B is the current mainstream first-line drug for HCC, there is no clinical trial using A+B as the control group as of date. A previous meta-analysis showed lenvatinib monotherapy achieved superior PFS compared to sorafenib, while no significant difference was observed in OS [19]. A recently published study used the network meta-analysis method to indirectly compare novel first-line systemic agents from randomized controlled trials. Fulgenzi et al. used sorafenib as a reference to perform analyses and found both A+B (HR 0.66; 95% CI 0.52–0.84) and L+P (HR 0.77; 95% CI 0.62–0.97) similarly reduced the risk of death. As for PFS, they found L+P had the best performance in reducing the risk of progression (L+P: HR 0.52; 95% CI 0.35–0.77; A+B: HR 0.63; 95% CI 0.44–0.91) among several combination therapies using sorafenib as the comparison reference [20]. Our present study supported that either an A+B or L+P combination regimen could be a reasonable choice for advanced HCC patients. This is also the first direct comparison study to demonstrate the comparable treatment efficacy of these two regimens in patients with advanced HCC.

Our study showed survival outcomes coincide with several other real-world studies that investigated lenvatinib-based ICI combinations. Our study has two unique features: our cohort focused solely on first-line systemic L+P treatment and used A+B as the active comparator. From six real-world studies that all included Asian patients [21,22,23,24,25,26], lenvatinib plus ICI achieved a median PFS of 6.6–10.6 months and a median OS of 11.4–18.4 months. Yang et al. retrospectively collected the largest cohort of 378 patients with unresectable HCC treated with lenvatinib plus PD-1 inhibitors as first-line or subsequent therapy [21]. With 18.3% of patients treated with L+P, they reported there was no significant difference in survival outcome between L+P and lenvatinib plus other PD-1 inhibitors. Our study is the first to use A+B as an active comparator when comparing with all the prior real-world studies, which further supported L+P treatment as one of the effective regimens for HCC.

Our study found factors such as Vp4 invasion and baseline liver function may easily interfere with patient survival outcomes. The HCC patients with portal vein tumor thrombosis usually had poor OS due to increased risk of worsening liver function, tumor spread, and portal hypertension-associated complications [27]. The patients with main portal vein invasion (Vp4) were excluded in both the REFLECT trial and the LEAP-002 trial [5,13]. However, lenvatinib could be considered in patients with Vp4 thrombosis and preserved liver function in some real-world studies [28,29]. An exploratory analysis of the updated IMbrave150 cohort supported the use of A+B in patients with Vp4 thrombosis [30]. As for lenvatinib-based combination therapy, Sun et al. retrospectively collected 84 HCC patients, with 30 patients having Vp4 thrombosis. They found that Vp4 portal vein thrombosis did not significantly affect ORR, PFS, and OS and reported no significant correlation between any kind of adverse events and Vp4 thrombosis [25]. Our study included 30 (24.8%) patients with Vp4 thrombosis and reported worsened OS in the multivariate analysis. The poorer survival outcomes observed among Vp4 patients in the entire cohort may be associated with the greater number of high-grade TRAEs in the A+B group with Vp4. Hence, managing treatment-related adverse events is crucial in the era of combination therapy. However, if patients still had a high risk of gastrointestinal bleeding after appropriate endoscopic treatment or had contraindications to A+B, L+P could be an alternative therapy based on our results. Recently, a phase 2 study demonstrated that the combination of immunotherapy and radiotherapy showed promising results as a first-line treatment for HCC with portal vein tumor thrombosis [31]. We still need more clinical trials to guide the treatment of patients with high-grade portal vein thrombosis. 

Our study showed Child–Pugh B liver dysfunction was a negative prognostic factor on PFS. Patients without preserved liver function were often excluded from landmark clinical trials, which led to a lack of high-quality evidence in the systemic therapy of HCC. Current guidelines suggest individualized, case-by-case evaluation, in which selected patients with Child–Pugh B liver function may be offered a single systemic agent [2,11]. Whether combination therapy is beneficial for patients with Child–Pugh B liver dysfunction is still debatable. Both Wu’s pure L+P cohort and Yang’s large cohort revealed patients with Child–Pugh B liver function have poor OS [21,24]. As for A+B, one recent meta-analysis revealed that patients with Child–Pugh B dysfunction had moderate treatment efficacy but may suffer more grade ≥3 adverse events [32]. Nonetheless, we still need more evidence to support that combination therapy is safe and effective for patients with advanced HCC.

There were several limitations in our study. First, our study was a retrospective study. While propensity score matching was utilized to reduce the impact of confounding factors between the two treatment groups, the potential bias still existed. Additionally, treatment-related adverse events may be underestimated due to recall bias. Second, most patients in our study were diagnosed with HCC secondary to viral hepatitis, particularly HBV-related HCC. Our study could not be extrapolated to populations with non-viral HCC as the primary etiology. Third, a significant number of patients did not receive the recommended dose of pembrolizumab due to economic constraints or adverse events during treatment. Therefore, our study results could not be directly compared with LEAP-002. 

## 5. Conclusions

In this real-world study, lenvatinib plus pembrolizumab (L+P) and atezolizumab plus bevacizumab (A+B) in the first-line setting provided comparable survival benefits and safety profiles in patients with unresectable HCC. Our findings support that either regimen could be a reasonable choice for advanced HCC patients, even in those with high tumor burden.

## Figures and Tables

**Table 1:** Baseline characteristics of the patients receiving combination therapy for unresectable HCCs.

	Entire	L+P	Pre-Matched A+B	*p* Value
Number	121	37	84	
Age (mean (SD))	61.50 (12.27)	59.01 (12.78)	62.59 (11.96)	0.14
Sex: Male (%)	95 (78.5)	29 (78.4)	66 (78.6)	1
HBV (%)	83 (68.6)	26 (70.3)	57 (67.9)	0.96
HCV (%)	20 (16.7)	6 (16.2)	14 (16.9)	1
Viral etiology (%)	99 (81.8)	31 (83.8)	68 (81.0)	0.91
Child–Pugh A/B (%)	107/14 (88.4/11.6)	30/7 (81.1/18.9)	77/7 (91.7/8.3)	0.17
Baseline ALBI grade I/II+III (%)	48/73 (39.7/60.3)	14/23 (37.8/62.2)	34/50 (40.5/59.5)	0.94
BCLC C (%)	95 (78.5)	29 (78.4)	66 (78.6)	1
Out of up-to-seven criteria (%)	103 (85.1)	30 (81.1)	73 (86.9)	0.58
Macrovascular invasion (%)				0.28
No MVI	54 (44.6)	17 (45.9)	37 (44.0)	
Vp2 and Vp3 stage	37 (30.6)	14 (37.8)	23 (27.4)	
Vp4 stage	30 (24.8)	6 (16.2)	24 (28.6)	
Extrahepatic spread (%)	52 (43.0)	18 (48.6)	34 (40.5)	0.52
Platelets (mean (SD)), 1000/uL	221.88 (106.18)	222.09 (110.52)	221.80 (105.00)	0.99
INR (mean (SD))	1.17 (0.16)	1.17 (0.18)	1.18 (0.15)	0.86
AST (mean (SD)), U/L	84.00 (76.45)	98.94 (87.19)	77.60 (70.96)	0.16
ALT (mean (SD)), U/L	68.04 (62.28)	64.14 (43.65)	69.78 (69.16)	0.65
Albumin (mean (SD)), g/dL	3.76 (0.50)	3.76 (0.44)	3.76 (0.52)	1
Total bilirubin (mean (SD)), mg/dL	1.20 (1.61)	1.49 (2.64)	1.07 (0.82)	0.18
AFP (median [range]), ng/mL	555.40 [2.00, 333,387.70]	1030.20 [2.80, 252,632.80]	463.50 [2.00, 333,387.70]	0.14
AFP greater than 400 ng/mL (%)	65 (53.7)	21 (56.8)	44 (52.4)	0.8
Combine locoregional therapy	47 (38.8)	11 (29.7)	36 (42.9)	0.24

AFP alpha-fetoprotein, ALBI grade, albumin–bilirubin grade, AST aspartate aminotransferase, ALT alanine transaminase, BCLC Barcelona Clinic Liver Cancer classification, HBV hepatitis B virus, HCV hepatitis C virus, INR international normalized ratio, MVI microvascular invasion, SD standard deviation, VP portal vein invasion.

**Table 2:** Efficacy outcomes of our patients receiving L+P or A+B before and after propensity score matching.

	Entire (*n* = 121)	L+P (*n* = 37)	Pre-Matched A+B (*n* = 84)	*p* Value	Post-Matched (*n* = 74)	Matched A+B (*n* = 37)	*p* Value
Tumor response at the first evaluation (%)				0.93			0.52
CR	1 (0.8)	0 (0.0)	1 (1.2)		1 (1.4)	1 (2.7)	
PR	38 (31.4)	11 (29.7)	27 (32.1)		24 (32.4)	13 (35.1)	
SD	38 (31.4)	13 (35.1)	25 (29.8)		26 (35.1)	13 (35.1)	
PD	36 (29.8)	11 (29.7)	25 (29.8)		21 (28.4)	10 (27.0)	
No image evaluation	8 (6.6)	2 (5.4)	6 (7.1)		2 (2.7)	0 (0.0)	
ORR	32%	30%	33%	0.7	34%	38%	0.47
DCR	64%	65%	63%	0.85	69%	73%	0.46
Follow-up duration, months (median [range])	9.90 [1.00, 39.42]	9.39 [1.90, 39.42]	10.12 [1.00, 33.33]	0.9	10.12 [1.50, 39.42]	10.90 [1.50, 33.33]	0.67
Treatment duration, months (median [range])	3.00 [0.61, 32.58]	2.84 [0.68, 13.87]	3.17 [0.61, 32.58]	0.88	3.31 [0.68, 32.58]	4.07 [0.68, 32.58]	0.53
Mortality	57 (47.1%)	15 (40.5%)	42 (50.0%)	0.45	33 (44.6%)	18 (48.6%)	0.64
Cancer-related mortality	53 (43.8%)	15 (40.5%)	38 (45.2%)	0.78	32 (43.2%)	17 (45.9%)	0.81

CR complete response, DCR disease control rate, ORR objective response rate, OS overall survival, PD progressive disease, PR partial response, SD stable disease.

**Table 3:** Univariate and multivariate Cox analyses of the entire cohort for overall survival (a) and progression-free survival (b).

**(a)**					
**Variables**	**Contrast**	**Hazard Ratio (95% CI)**	***p* Value**	**Adjusted Hazard Ratio**	***p* Value**
Age	≥65 vs. <65	0.78 (0.44, 1.39)	0.40		
Sex	Male vs. Female	0.76 (0.41, 1.41)	0.38		
Systemic therapy	L+P vs. A+B	0.75 (0.41, 1.37)	0.35	0.79 (0.44, 1.45)	0.45
Viral etiology	Yes vs. No	0.91 (0.47, 1.76)	0.78		
Child–Pugh class	B vs. A	2.21 (1.11, 4.39)	0.02	1.67 (0.82, 3.44)	0.16
ALBI grade	II/III vs. I	1.90 (1.09, 3.31)	0.02		
BCLC	C vs. B	2.33 (1.09, 5.00)	0.03		
Seven criteria	Beyond vs. Within	1.78 (0.71, 4.46)	0.22		
Vp4 stage	Yes vs. No	2.32 (1.31, 4.09)	<0.01	2.08 (1.17, 3.72)	0.01
Extrahepatic spread	Yes vs. No	1.12 (0.66, 1.88)	0.68		
AFP > 400	Yes vs. No	1.77 (1.03, 3.04)	0.04	1.58 (0.90, 2.76)	0.11
Early AFP response	Yes vs. No	0.58 (0.33, 1.02)	0.06		
Combine LRT	Yes vs. No	0.82 (0.48, 1.40)	0.47		
**(b)**					
**Variables**	**Contrast**	**Hazard Ratio (95% CI)**	***p* Value**	**Adjusted Hazard Ratio**	***p* Value**
Age	≥65 vs. <65	0.65 (0.40, 1.08)	0.10		
Sex	Male vs. Female	1.15 (0.64, 2.06)	0.65		
Systemic therapy	L+P vs. A+B	0.92 (0.55, 1.53)	0.75		
Viral etiology	Yes vs. No	0.84 (0.47, 1.51)	0.57		
Child–Pugh class	B vs. A	2.10 (1.13, 3.93)	0.02	2.77 (1.45, 5.29)	<0.01
ALBI grade	II/III vs. I	1.54 (0.95, 2.50)	0.08		
BCLC	C vs. B	2.02 (1.05, 3.86)	0.03		
Seven criteria	Beyond vs. Within	1.34 (0.64, 2.80)	0.43		
Vp4 stage	Yes vs. No	1.58 (0.95, 2.64)	0.08		
Extrahepatic spread	Yes vs. No	1.01 (0.63, 1.60)	0.97		
AFP > 400	Yes vs. No	1.64 (1.02, 2.64)	0.04		
Early AFP response	Yes vs. No	0.40 (0.24, 0.68)	<0.001	0.40 (0.24, 0.67)	<0.001
Combine LRT	Yes vs. No	0.57 (0.35, 0.92)	0.02	0.56 (0.34, 0.92)	0.02

AFP alpha-fetoprotein, ALBI grade, albumin–bilirubin grade, BCLC Barcelona Clinic Liver Cancer classification, LRT locoregional therapy, VP portal vein invasion.

**Table 4:** Treatment-related adverse events.

	Lenvatinib Plus Pembrolizumab (*n* = 37)		Atezolizumab Plus Bevacizumab (*n* = 84)	
Adverse events, *n* (%)	Any grade	Grade 3 or above	Any grade	Grade 3 or above
Any adverse events	30 (81.1%)	10 (27.0%)	62 (73.8%)	16 (19.0%)
Diarrhea	9 (24.3%)	2 (5.4%)	7 (8.3%)	0 (0.0%)
Anorexia	6 (16.2%)	0 (0.0%)	10 (11.9%)	0 (0.0%)
Fatigue	4 (10.8%)	0 (0.0%)	7 (8.3%)	0 (0.0%)
Elevated aspartate aminotransferase	10 (27.0%)	1 (2.7%)	32 (38.1%)	3 (3.6%)
Elevated alanine transaminase	12 (32.4%)	1 (2.7%)	23 (27.4%)	1 (1.2%)
Rash	5 (13.5%)	1 (2.7%)	7 (8.3%)	1 (1.2%)
Esophageal variceal bleeding	1 (2.7%)	1 (2.7%)	9 (10.7%)	9 (10.7%)
Palmar-plantar erythrodysesthesia	8 (21.6%)	3 (8.1%)	0 (0.0%)	0 (0.0%)
Hypertension	5 (13.5%)	1 (2.7%)	9 (10.7%)	0 (0.0%)
Proteinuria	13 (35.1%)	1 (2.7%)	10 (11.9%)	0 (0.0%)
Adrenal insufficiency	0 (0.0%)	0 (0.0%)	4 (4.8%)	0 (0.0%)
Sepsis	2 (5.4%)	2 (5.4%)	2 (2.4%)	2 (2.4)
Nausea	1 (2.7%)	0 (0.0%)	2 (2.4%)	0 (0.0%)
Dysphonia	2 (5.4%)	0 (0.0%)	0 (0.0%)	0 (0.0%)
Pancreatitis	1 (2.7%)	0 (0.0%)	1 (1.2%)	0 (0.0%)
Acute kidney injury	0 (0.0%)	0 (0.0%)	1 (1.2%)	0 (0.0%)
Pneumonitis	1 (2.7%)	0 (0.0%)	0 (0.0%)	0 (0.0%)

## Data Availability

All data generated or analyzed during this study are included in this article. Further enquiries can be directed to the corresponding author.

## Supplementary Materials

The following supporting information can be downloaded at: https://www.mdpi.com/article/10.3390/cancers16203458/s1, Table S1: Baseline characteristics of the patients receiving combination therapy for unresectable HCCs after propensity score matching, Table S2: Univariate Cox analyses for overall survival and progression-free survival in the patients receiving lenvatinib plus pembrolizumab, Figure S1: Effectiveness of propensity score matching (a) jitter plot (b) histogram (c) Love plot, Figure S2: Kaplan-Meier curves of (a) overall survival (OS) of the patients with Child-Pugh A liver function; (b) progression-free survival (PFS) of the patients with Child-Pugh A liver function.
