# Supplementary material for: Comparing Lenvatinib/Pembrolizumab with Atezolizumab/Bevacizumab in Unresectable Hepatocellular Carcinoma: A Real-World Experience with Propensity Score Matching Analysis
Source: Cancers (Basel). 2024 Oct 12;16(20):3458. doi: 10.3390/cancers16203458 (PMC11506658; doi:10.3390/cancers16203458)
Supplement: Supplementary file 1 [file cancers-16-03458-s001.zip › cancers-3238384-supplementary.pdf]

Comparing Lenvatinib/Pembrolizumab with Atezolizumab/Bevacizumab in Unresectable Hepatocellular Carcinoma: A Real-World Experience with Propensity Score Matching Analysis

Yu-Chun Hsu [1,2], Po-Ting Lin [1,2,3], Wei Teng [1,2], Yi-Chung Hsieh [1,2], Wei-Ting Chen [1,2], Chung-Wei Su [1,2], Ching-Ting Wang [2,4], Pei-Mei Chai [2,4], Chen-Chun Lin [2,5], Chun-Yen Lin [1,2,*] and Shi-Ming Lin [1,2,*]

1 Department of Gastroenterology and Hepatology, Chang Gung Memorial Hospital, Linkou Branch, Taoyuan 333, Taiwan
2 College of Medicine, Chang Gung University, Taoyuan 333, Taiwan
3 Graduate Institute of Clinical Medical Sciences, College of Medicine, Chang Gung University, Taoyuan 333, Taiwan
4 Department of Nursing, Chang Gung Memorial Hospital, Linkou Branch, Taoyuan 333, Taiwan
5 Department of Gastroenterology and Hepatology, New Taipei Municipal Tucheng Hospital, New Taipei 236, Taiwan
* Correspondence: chunyenlin@gmail.com (C.-Y.L.); lsmpaicyto@gmail.com (S.-M.L.)

Table S1. Baseline characteristics of the patients receiving combination therapy for unresectable HCCs after propensity score matching

	Matched patients	L+P	Matched A+B	P value
Number	74	37	37	
Age (mean (SD))	58.29 (11.94)	59.01 (12.78)	57.56 (11.16)	0.6
Sex: Male (%)	58 (78.4)	29 (78.4)	29 (78.4)	1
HBV (%)	56 (75.7)	26 (70.3)	30 (81.1)	0.42
HCV (%)	9 (12.2)	6 (16.2)	3 (8.1)	0.48
Viral etiology (%)	63 (85.1)	31 (83.8)	32 (86.5)	1
Child-Pugh A/B (%)	64/10 (86.5/13.5)	30/7 (81.8/18.9)	34/3 (91.9/8.1)	0.31
Baseline ALBI grade I/II+III (%)	29/45 (39.2/60.8) (39.2/60.8)	14/23 (37.8/62.2)	15/22 (40.5/59.5)	1
BCLC C (%)	59 (79.7)	29 (78.4)	30 (81.1)	1
Out of up-to-seven criteria (%)	60 (81.1)	30 (81.1)	30 (81.1)	1
Macrovascular invasion (%)				0.62
No MVI	34 (45.9)	17 (45.9)	17 (45.9)	
Vp2 and Vp3 stage	25 (33.8)	14 (37.8)	11 (29.7)	
Vp4 stage	15 (20.3)	6 (16.2)	9 (24.3)	
Extrahepatic spread (%)	36 (48.6)	18 (48.6)	18 (48.6)	1
Platelets (mean (SD)), 1000/uL	225.68 (118.71)	222.09 (110.52)	229.08 (127.41)	0.8
INR (mean (SD))	1.18 (0.16)	1.17 (0.18)	1.19 (0.14)	0.52
AST (mean (SD)), U/L	93.40 (85.25)	98.94 (87.19)	88.00 (84.17)	0.59
ALT (mean (SD)), U/L	71.96 (67.80)	64.14 (43.65)	79.78 (85.39)	0.32
Albumin (mean (SD)), g/dL	3.77 (0.53)	3.76 (0.44)	3.78 (0.59)	0.89
Total bilirubin (mean (SD)), mg/dL	1.33 (1.99)	1.49 (2.64)	1.16 (0.99)	0.48
AFP (median [range]), ng/ml	1158.45 [2.00, 333387.70]	1030.20 [2.80, 252632.80]	1169.40 [2.00, 333387.70]	0.66
AFP greater than 400 ng/ml (%)	44 (59.5)	21 (56.8)	23 (62.2)	0.81
Combine locoregional therapy	26 (35.1)	11 (29.7)	15 (40.5)	0.47

AFP alpha-fetoprotein, ALBI grade, albumin-bilirubin grade, AST aspartate

aminotransferase, ALT alanine transaminase, BCLC Barcelona Clinic Liver Cancer classification, HBV hepatitis B virus, HCV hepatitis C virus, INR international normalized ratio, MVI microvascular invasion, SD standard deviation, VP portal vein invasion.

Figure S1. Effectiveness of propensity score matching (a) jitter plot (b) histogram (c) Love plot.

(a) Jitter plot

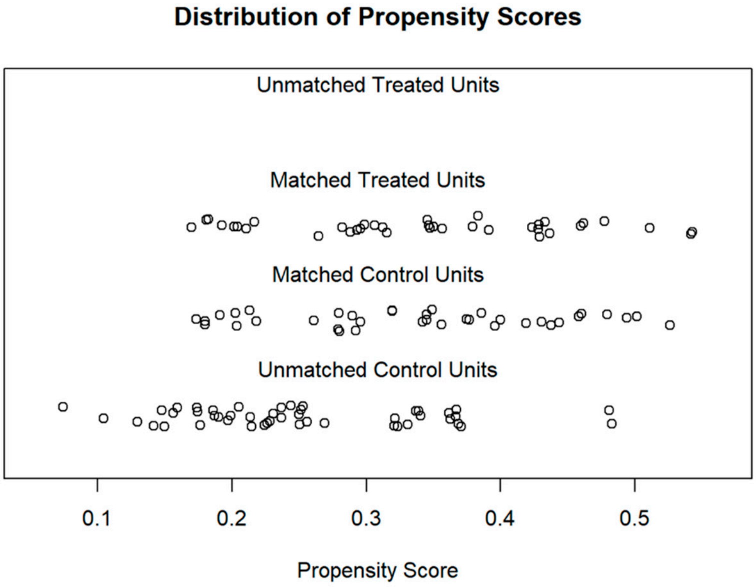

(b) Histogram

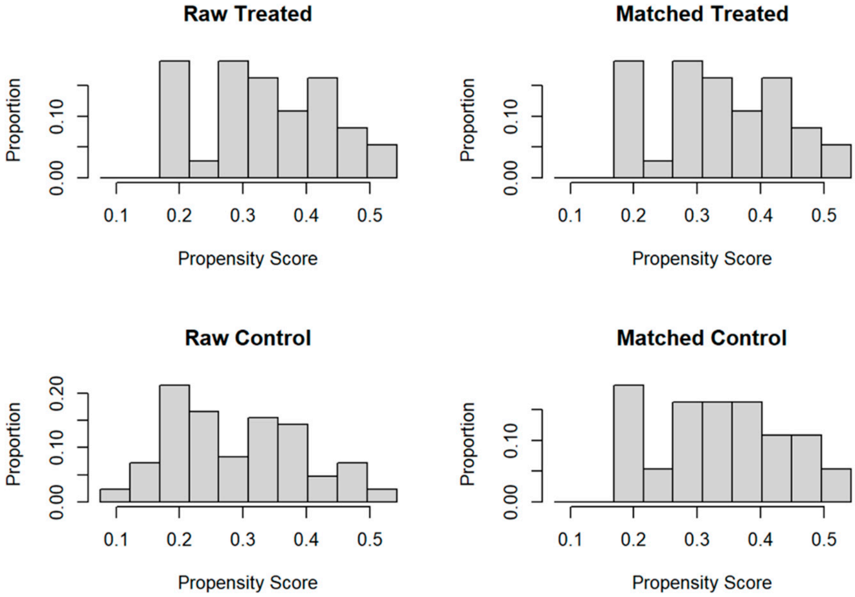

(c) Love plot

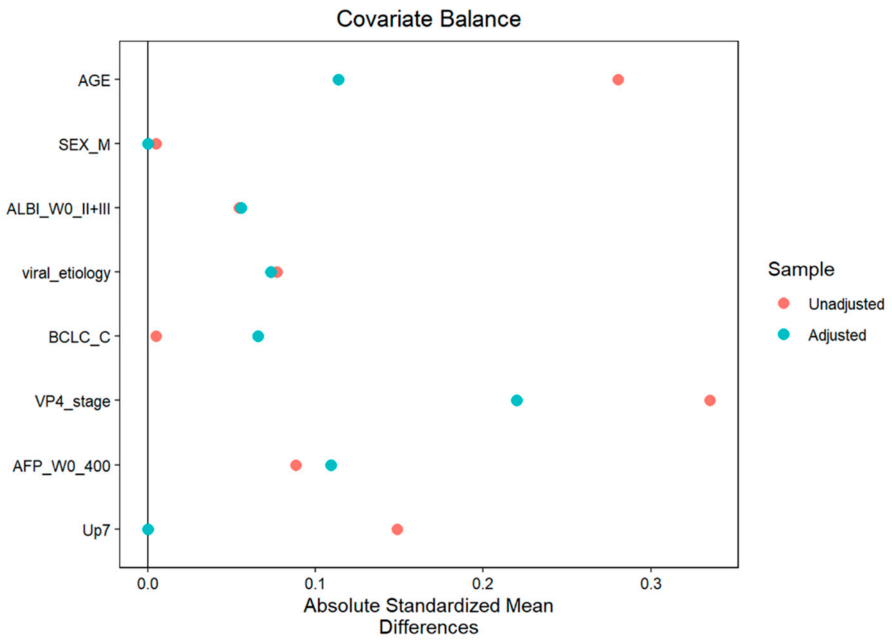

Figure S2. Kaplan-Meier curves of (a) overall survival (OS) of the patients with Child-Pugh A liver function; (b) progression-free survival (PFS) of the patients with Child-Pugh A liver function.

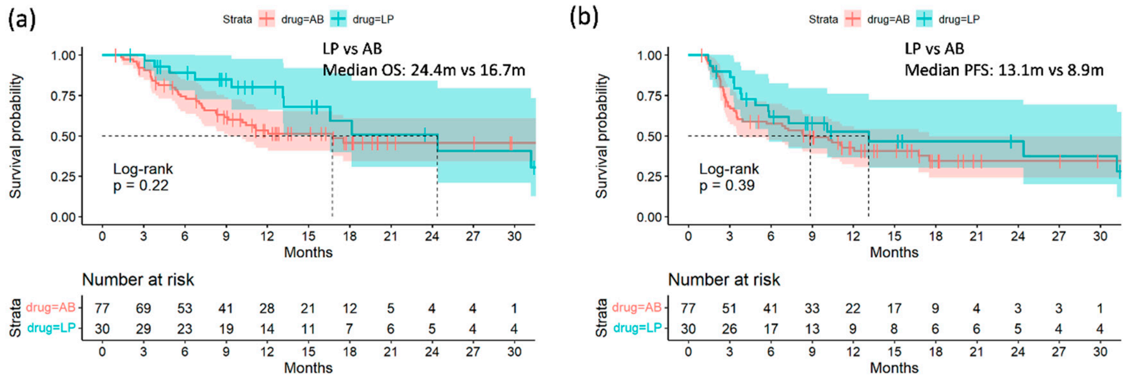

Table S2. Univariate Cox analyses for overall survival and progression-free survival in the patients receiving lenvatinib plus pembrolizumab

Variables	Contrast	Hazard ratio (95% CI) for OS	P value	Hazard ratio (95% CI) for PFS	P value
Age	>= 65 vs. < 65	0.95 (0.30, 3.00)	0.925	0.76 (0.30, 1.96)	0.574
Sex	Male vs. Female	3.88 (0.51, 29.61)	0.191	3.34 (0.78, 14.30)	0.105
Viral etiology	Yes vs. No	0.46 (0.15, 1.47)	0.192	0.94 (0.32, 2.78)	0.908
Child-Pugh class	B vs. A	5.23 (1.50, 18.19)	0.009	4.22 (1.56, 11.38)	0.004
ALBI grade	II/III vs. I	1.39 (0.47, 4.10)	0.546	1.11 (0.46, 2.66)	0.813
BCLC	C vs. B	2.79 (0.77, 10.99)	0.141	3.53 (0.97, 12.90)	0.056
Seven criteria	Beyond vs. Within	2.91 (0.38, 22.19)	0.303	2.13 (0.50, 9.16)	0.307
Vp4 stage	Yes vs. No	1.04 (0.23, 4.76)	0.964	1.38 (0.46, 4.15)	0.566
Extrahepatic spread	Yes vs. No	1.94 (0.68, 5.58)	0.217	1.64 (0.70, 3.84)	0.253
AFP > 400	Yes vs. No	2.32 (0.79, 6.87)	0.128	1.77 (0.74, 4.24)	0.198
Early AFP response	Yes vs. No	0.59 (0.21, 1.65)	0.318	0.29 (0.12, 0.71)	0.007
Combine LRT	Yes vs. No	0.93 (0.32, 2.68)	0.896	0.59 (0.23, 1.53)	0.282

AFP alpha-fetoprotein, ALBI grade, albumin-bilirubin grade, BCLC Barcelona Clinic Liver Cancer classification, LRT locoregional therapy, VP portal vein invasion.
